# Myeloablative autologous haematopoietic stem cell transplantation resets the B cell repertoire to a more naïve state in patients with systemic sclerosis

**DOI:** 10.1136/ard-2021-221925

**Published:** 2022-10-14

**Authors:** Julia Z Adamska, Amin Zia, Michelle S Bloom, Leslie J Crofford, Daniel E Furst, Ellen Goldmuntz, Lynette Keyes-Elstein, Maureen D Mayes, Peter McSweeney, Richard A Nash, Ashley Pinckney, Beverly Welch, Zelda Z Love, Keith M Sullivan, William Robinson

**Affiliations:** 1 Division of Immunology and Rheumatology, Department of Medicine, Stanford University, Stanford, California, USA; 2 VA Palo Alto Health Care System, Palo Alto, California, USA; 3 Division of Rheumatology and Immunology, Vanderbilt University, Nashville, Tennessee, USA; 4 Rheumatology, Univ of Cal at Los Angeles, Los Angeles, California, USA; 5 Division of Allergy, Immunology, and Transplantation, NIH/NIAID, Bethesda, Maryland, USA; 6 Rho Federal Systems Division, Durham, North Carolina, USA; 7 Rheumatology and Clinical Immunogenetics, The University of Texas Health Science Center Houston Medical School, Houston, Texas, USA; 8 Rocky Mountain Blood and Marrow Transplant Program, Colorado Blood Cancer Institute, Denver, Colorado, USA; 9 Department of Medicine, Duke University Health System, Durham, North Carolina, USA

**Keywords:** Systemic Sclerosis, B-Lymphocytes, Immune System Diseases

## Abstract

**Objectives:**

Myeloablative autologous haematopoietic stem cell transplant (HSCT) was recently demonstrated to provide significant benefit over cyclophosphamide (CYC) in the treatment of diffuse cutaneous systemic sclerosis (dcSSc) in the Scleroderma: Cyclophosphamide or Transplantation (SCOT) trial. As dysregulation of the B cell compartment has previously been described in dcSSc, we sought to gain insight into the effects of myeloablative autologous HSCT as compared with CYC.

**Methods:**

We sequenced the peripheral blood immunoglobulin heavy chain (IGH) repertoires in patients with dcSSc enrolled in the SCOT trial.

**Results:**

Myeloablative autologous HSCT was associated with a sustained increase in IgM isotype antibodies bearing a low mutation rate. Clonal expression was reduced in IGH repertoires following myeloablative autologous HSCT. Additionally, we identified a underusage of immunoglobulin heavy chain V gene 5–51 in patients with dcSSc, and usage normalised following myeloablative autologous HSCT but not CYC treatment.

**Conclusions:**

Together, these findings suggest that myeloablative autologous HSCT resets the IGH repertoire to a more naïve state characterised by IgM-expressing B cells, providing a possible mechanism for the elimination of pathogenic B cells that may contribute to the benefit of HSCT over CYC in the treatment of dcSSc.

**WHAT IS ALREADY KNOWN ABOUT THIS SUBJECT**

- Myeloablative autologous haematopoietic stem cell transplant (HSCT) was recently demonstrated to provide benefit in the treatment of diffuse cutaneous systemic sclerosis (dcSSc).

**WHAT THIS STUDY ADDS**

- The results of this study suggest that, as compared with treatment with cyclophosphamide (CYC), myeloablative autologous HSCT resets the immunoglobulin heavy chain repertoire to a more naïve state characterised by IgM-expressing B cells, thereby providing a possible mechanism for the elimination of pathogenic B cells that may contribute to the benefit of HSCT in the treatment of dcSSc.

**HOW THIS STUDY MIGHT AFFECT RESEARCH, PRACTICE OR POLICY**

- This study provides new insights into the potential mechanism by which myeloablative autologous HSCT provides benefit and superiority as compared to CYC in the treatment of dcSSc, which adds to the scientific rational for use of HSCT to treat dcSSc.

## Introduction

Diffuse cutaneous systemic sclerosis (dcSSc) is a chronic, often fatal, autoimmune disease characterised by vascular damage, and excessive extracellular matrix accumulation in the skin and visceral organs and is associated with dysregulated B and T cells. Studies have shown that impaired function of regulatory T cells is associated with severity of dcSSc^1^ and that T cell receptor diversity is decreased in dcSSc.^2^ Further, increased B cell activation^3–5^ and autoantibody production,^6^ along with decreased regulatory B cell (Breg) and impaired interleukin-10 production have been associated with dcSSc.^7–10^

Currently, there are few effective treatments for dcSSc. Cyclophosphamide (CYC), an alkylating agent used to treat many autoimmune diseases and cancers, yields limited short-term benefit.^11–14^ Non-myeloablative haematopoietic stem cell transplantation (HSCT)^15–18^ resulted in increased benefit compared with CYC, demonstrating the utility of transplantation therapies. However, there are concerns about the safety and durability of non-myeloablative treatments.^18–20^

Recently, the Scleroderma: Cyclophosphamide or Transplantation (SCOT) clinical trial demonstrated that myeloablative CD34-selected autologous HSCT provided significant benefit over monthly CYC treatment in individuals with dcSSc.^21^ Further, another study demonstrated that HSCT resulted in decreased peripheral B cell replication, compensated by increased output of newly generated bone marrow derived naïve B cells and Bregs.^8^

CYC depletes certain populations of T cells^22^ and suppresses B cells.^23^ Additionally, CYC is immunosuppressive by virtue of toxicity to lymphocytes, with less impact on haematopoietic stem cells (HSCs). In contrast, myeloablative autologous HSCT treatment depletes both lymphocytes and HSCs by total body irradiation, with reconstitution of these population through CD34-selected HSC autograft. Thus, myeloablative autologous HSCT treatment aims to deplete the autoimmune repertoire and re-establish a new, auto-tolerant immune system.

In this study, we analysed a subset of samples from the SCOT trial to determine the differential effects of myeloablative autologous HSCT as compared with CYC treatment on the B cell antibody repertoire. The SCOT trial directly compared myeloablative CD34^+^ selected autologous HSCT therapy, to high-dose immunosuppressive therapy with CYC. The SCOT study was designed to assess the effectiveness of HSCT versus high-immunosuppressive therapy by CYC. In the HSCT group, irradiation and CYC (120 mg/kg of body weight) were used once, at the start of treatment, to eliminate lymphocytes and pathogenic B cells prior to transplantation. Further details regarding the patient population and treatment regimens are available in the primary manuscript describing the SCOT trial.^21^ By sequencing bulk blood RNA, we identify differences in immunoglobulin heavy chain V gene (IGHV gene) usage that differentiate patients with dcSSc from healthy controls. Through sequencing IGHV genes, we demonstrated that increases in IgM antibodies following myeloablative autologous HSCT contribute to the decreased mutation rates observed in these individuals. Finally, we demonstrated that following myeloablative autologous HSCT, individuals experienced a decrease in clonality in their immunoglobulin heavy chain (IGH) repertories. Together, these findings indicate that in contrast to CYC treatment, myeloablative autologous HSCT results in a repopulation of un-class switched IgM-expressing B cells with restricted clonal expressions, which may prevent the expansion of potentially pathogenic B cell.

## Methods

### Human samples

We studied RNA samples from a subset of the individuals included in the full SCOT clinical trial.^21^ RNA samples were provided to Stanford University by the contract research service Rho (http://www.rhoworld.com/) as barcoded samples with no identifiers. The cohort analysed is comprised of 15 recipients of HSCT and 16 recipients of CYC, along with 15 healthy controls with only baseline data. Patient demographic data can be found in table 1. The mean age for each group is: HSCT=42 y/o, CYC=45 y/o and HC=44 y/o.

**Table 1:** Patient samples and demographic data

Patient samples						Demographic data					
Patient ID	Treatment	M0	M26	M38	M48						
SCOT2	HSCT	**X**	**X**	**X**	**X**						
SCOT3	HSCT	**X**				**Ethnic categories from HSCT group**					
SCOT21	HSCT	**X**	**X**	**X**		**Racial categories**	**Not Hispanic or Latino**		**Hispanic or Latino**		**Total**
SCOT27	HSCT	**X**	**X**	**X**	**X**		**Female**	**Male**	**Female**	**Male**	
SCOT32	HSCT	**X**	**X**			**Asian**	1	0	0	0	1
SCOT33	HSCT	**X**		**X**	**X**						
SCOT37	HSCT	**X**				**Black or African American**	0	2	0	0	2
SCOT42	HSCT	**X**	**X**		**X**						
SCOT45	HSCT	**X**		**X**	**X**	**White**	4	7	1	0	12
SCOT48	HSCT	**X**	**X**	**X**	**X**						
SCOT51	HSCT	**X**				**Multiple races**	0	0	0	0	0
SCOT53	HSCT	**X**	**X**	**X**							
SCOT58	HSCT	**X**				**Total**	5	9	1	0	15
SCOT71	HSCT			**X**	**X**						
SCOT74	HSCT	**X**	**X**	**X**	**X**						
SCOT5	CYC	**X**	**X**	**X**							
SCOT8	CYC	**X**				**Ethnic categories from CYC group**					
SCOT9	CYC	**X**	**X**	**X**	**X**	**Racial categories**	**Not Hispanic or Latino**		**Hispanic or Latino**		**Total**
SCOT12	CYC	**X**					**Female**	**Male**	**Female**	**Male**	
SCOT18	CYC	**X**				**Asian**	0	0	0	0	0
SCOT25	CYC	**X**	**X**	**X**	**X**						
SCOT26	CYC	**X**	**X**			**Black or African American**	2	0	0	0	2
SCOT41	CYC	**X**	**X**	**X**	**X**						
SCOT43	CYC	**X**	**X**	**X**		**White**	8	4	1	0	13
SCOT47	CYC	**X**	**X**	**X**							
SCOT50	CYC		**X**	**X**	**X**	**Multiple races**	1	0	0	0	1
SCOT63	CYC	**X**	**X**		**X**						
SCOT64	CYC	**X**				**Total**	11	4	1	0	16
SCOT67	CYC	**X**	**X**	**X**	**X**						
SCOT70	CYC		**X**								
SCOT73	CYC	**X**	**X**	**X**	**X**						
HC1	Healthy	**X**									
HC2	Healthy	**X**				**Ethnic categories from HC group**					
HC3	Healthy	**X**				**Racial categories**	**Not Hispanic or Latino**		**Hispanic or Latino**		**Total**
HC4	Healthy	**X**					**Female**	**Male**	**Female**	**Male**	
HC5	Healthy	**X**				**Asian**	0	0	0	0	0
HC6	Healthy	**X**									
HC7	Healthy	**X**				**Black or African American**	3	0	0	0	3
HC8	Healthy	**X**									
HC9	Healthy	**X**				**White**	6	6	0	0	12
HC10	Healthy	**X**									
HC11	Healthy	**X**				**Multiple races**	0	0	0	0	0
HC12	Healthy	**X**									
HC13	Healthy	**X**				**Total**	9	6	0	0	15
HC14	Healthy	**X**									
HC15	Healthy	**X**									

CYC, cyclophosphamide; HC, healthy control; HSCT, haematopoietic stem cell transplant; SCOT, Scleroderma: Cyclophosphamide or Transplantation.

### Experimental setup and IGH sequencing

Bulk RNA was previously extracted from Tempus tubes collected at baseline/pre-treatment, 26 months, 38 months and 48 months after initiation of treatment and at a single time point for healthy controls, as previously described.^24^ The healthy controls were matched for age and gender (online supplemental table1). Samples were spread out over three libraries and sequenced on the HiSeq2500 (325×275). All timepoints from the same patient were included in the same library. To minimise batch effects, each run included a mix of samples from the transplant (14–17), CYC (15–17) and healthy control (5–6) groups. RT/PCRs/library prep were performed using our standardised protocols over a couple week period and submitted at the same time for sequencing. We performed sequencing of the IGH variable, diversity and joining (VDJ) region adapted from Turchaninova *et al* 25 and as described.^26^ Each starting IGH RNA was tagged with a unique molecular identifier (UMI). The cDNA was amplified using nested primers, and sequenced using an asymmetrical paired end 325×275 cycle run on the Illumina HiSeq 2500.

10.1136/ard-2021-221925.supp1 Supplementary data

### Bioinformatics analysis

Fastq files were demultiplexed using Illumina CASAVA/bcl2fastq pipeline. Demultiplexed files were processed using MIGEC software,^27^ to identify UMIs and establish a consensus sequence, and embedded UMIs used to correct for PCR errors. Paired-end sequences were then combined using MITOOLS^28^ to obtain single long sequence reads, aligned to germline Ig loci using MiXCR,^28^ and features of the sequenced IGH locus analysed. Unsupervised hierarchical clustering was performed in R using the ‘pheatmap’ package. Samples that returned fewer than 1000 sequences aligned to IGH or that had greater than 10% of the repertoire consisting of an undetermined isotype were omitted. This resulted in the exclusion of five samples.

### Statistical analysis

Significance analysis of microarrays (SAM) was performed as described.^29^ Use of false discovery rates (FDRs) obviates the need to adjust for multiple comparisons. Comparisons with FDR(q)<0.1 were considered significant. Treatment groups were compared using an unpaired t-test, while observations for the same patient over time compared using a paired t-test. The Simpson Diversity Index (SI) was used as a quantitative measure of IGH sequence variability, and reflects the diversity of IGH sequences present in a dataset while accounting for their phylogenetic relations. The SI is at its minimum (1/N where N is the number of IGH sequences) when all IGH sequences are equally abundant, and increases when the population becomes heterogeneous.^30^

## Results

### HSCT is associated with longitudinal increases in percentage of IgM isotype and decreases in IgM-encoded somatic hypermutation levels

To analyse the different effects of myeloablative autologous HSCT and CYC treatment on B cell repertoire dynamics in individuals with dcSSc, we performed bulk RNA sequencing of the IGH from peripheral blood mononuclear cells (PBMCs) derived from 15 individuals undergoing HSCT and 16 individuals undergoing CYC treatment at baseline (prior to treatment) and at 26 months, 38 months and 48 months post-initiation of treatment. Patient demographic data were comparable between treatment groups. Due to the severity the disease, patients with dcSSc donated samples at various times during the study (table 1). There was no difference at baseline in the clinical characteristics between the two treatment groups.

**Figure 1:**
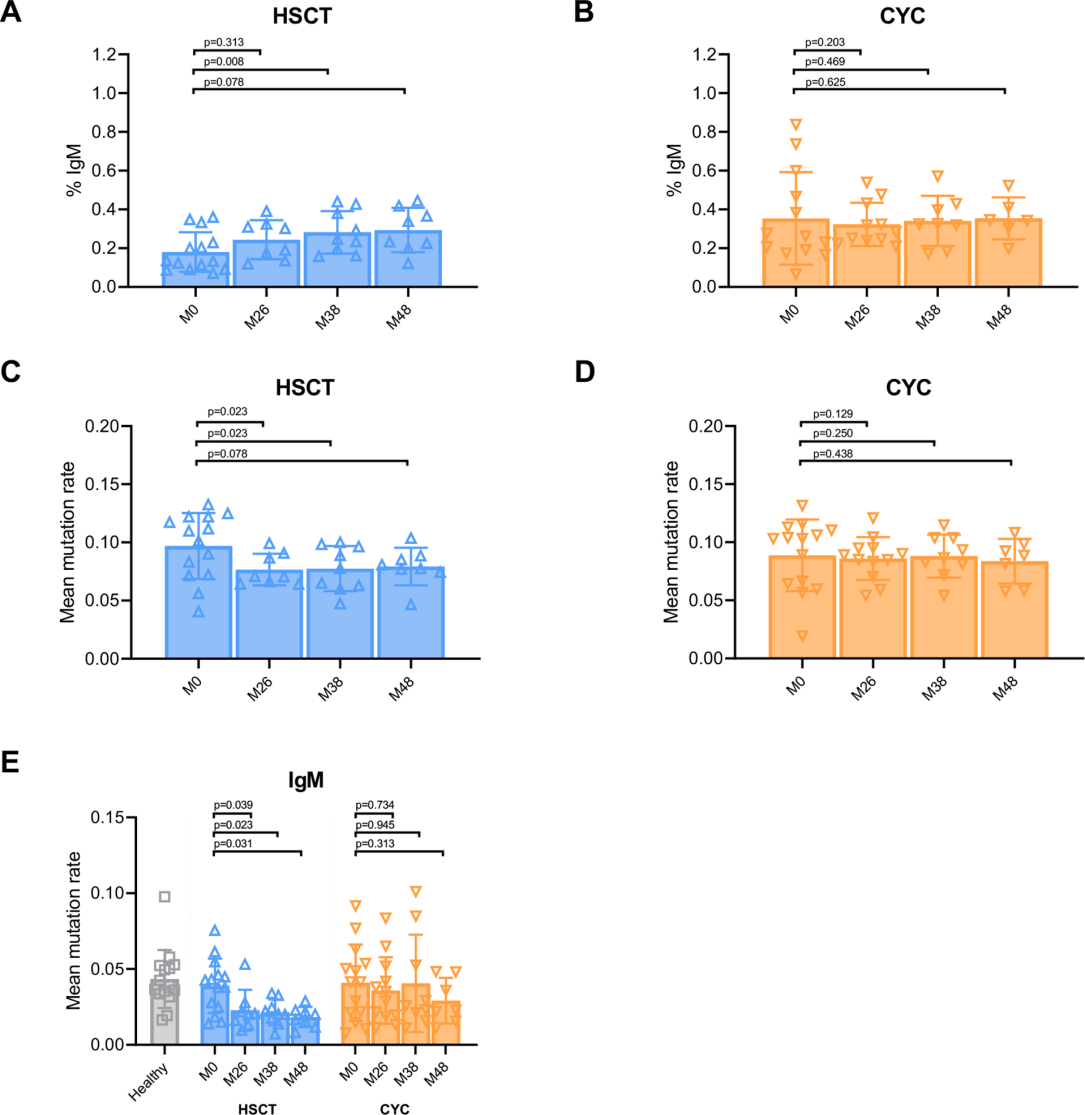
IgM-encoded decreases in somatic hypermutation levels following HSCT. (A, B) Per cent of repertoire comprised of IgM in individuals with dcSSc at baseline (M0) and 26, 38 and 48 months following either HSCT (A) or CYC treatment (B). (C, D) Quantification of mean somatic hypermutation rates in individuals with dcSSc at baseline and at 26, 38 and 48 months following either HSCT (C) or CYC treatment (D). (E) Quantification of mean somatic hypermutation rates for the fraction of each repertoire comprised of IgM in healthy individuals at baseline and in individuals with dcSSc at baseline and at 26, 38 and 48 months following either HSCT or CYC treatment. Data represent mean±SD, by paired t-test. CYC, cyclophosphamide; dcSSc, diffuse cutaneous systemic sclerosis; HSCT, haematopoietic stem cell transplant.

We analysed the effects of myeloablative autologous HSCT or CYC treatment on isotype composition of the IGH repertoires by calculating the percent of each repertoire comprised of either IgA, IgG or IgM at baseline and at 26, 38 and 48 months post-initiation of treatment. Comparison of the repertoires of both healthy and dcSSc individuals at baseline revealed no significant differences in the percent of repertoires comprised of either IgA, IgG or IgM (online supplemental figure 1A). Longitudinal analysis revealed that individuals whom underwent HSCT had significant increases in the percent of the repertoire comprised of IgM at 38 and 48 months after initial treatment (M0; figure 1A). In contrast, no significant differences in the percentage of repertoires comprised of IgM were seen in individuals following CYC treatment as compared with baseline (figure 1B). Further, no significant differences in the average per cent repertoire comprised of IgG or IgA were seen following either CYC or HSCT treatments as compared with baseline (online supplemental figure 1B).

10.1136/ard-2021-221925.supp2 Supplementary data

We next investigated the effects of HSCT and CYC treatments on IGH mutation rates. We determined mutation rates for both healthy individuals and those with dcSSc at baseline and at 26, 38 and 48 months post-initiation of treatment by identifying the number of mismatches in the variable region as compared with the corresponding areas of germline loci and normalised to the total size of the region. Mutation rates were averaged across all sequences in a sample to obtain the mean mutation rate. Comparison of the mean mutation rates between healthy individuals and individuals with dcSSc at baseline demonstrated no significant differences between the two groups (online supplemental figure 1C). Whereas significant decreases in mean mutation rates were observed at 26, 38 and 48 months following HSCT as compared with baseline, no significant differences were observed following CYC treatment (figure 1C and D).

Based on our finding that the IgM fraction of repertoires was expanded following HSCT, we analysed whether the changes in mutation rate following HSCT could be attributed to a single isotype. We determined the mean mutation rate at each timepoint following either HSCT or CYC treatment, for each isotype. We found that the mean mutation rates in the IgM fraction of the repertoires of individuals that underwent HSCT significantly decreased in months 26, 38 and 48 following treatment as compared with baseline (figure 1E). In contrast, no significant changes were seen in the mean mutation rate of the IgM fraction following treatment with CYC (figure 1E). Further, significant decreases in mutation rate were only observed in the IgA fraction 26 months following HSCT and in the IgG fraction 26 months following CYC treatment (online supplemental figure 1D). Together, these findings suggest that the expansion of B cells encoding IgM antibodies occurred following HSCT and is the main contributor to the decrease in mutations observed following HSCT.

### IGH repertoires exhibit decreases in clonality following HSCT

**Figure 2:**
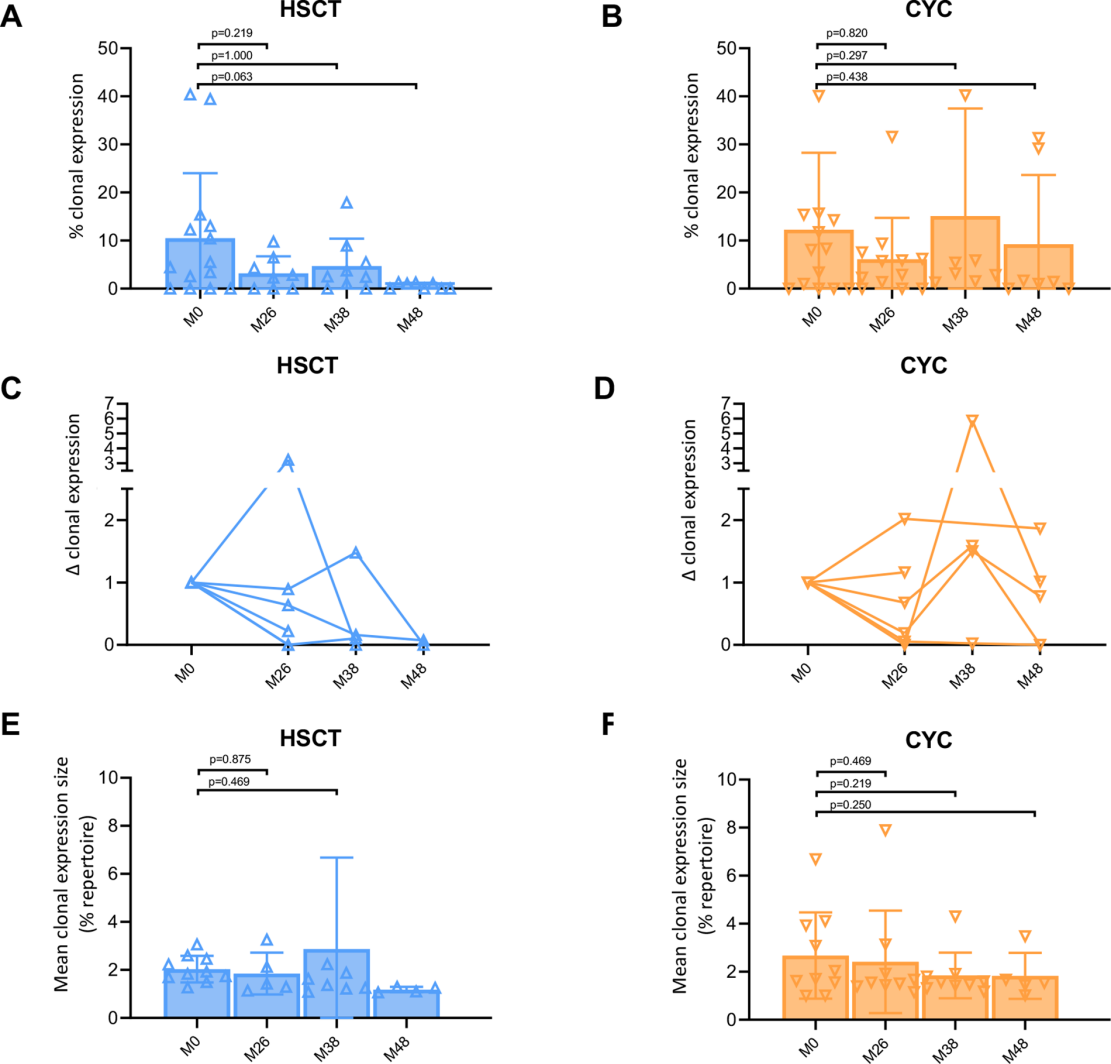
IGH repertoires exhibit decreases in clonality following HSCT. (A, B) Quantification of the per cent clonal expressions for repertoires from individuals with dcSSc at baseline (M0) and 26, 38 and 48 months following treatment with either HSCT (A) or CYC (B). (C) and (D) Changes in clonal expressions were assessed by normalising repertoire clonality at 26, 38 or 48 months following either HSCT (C) or CYC treatment (D) to clonality at baseline in five dcSSc individuals for which baseline and at least one additional consecutive timepoint sample was available. Lines connect data points from the same individual. (E) and (F) Clonal expression sizes were calculated as percentages of total repertoire size. Represented as quantification of mean clonal expression size in the repertoires of individuals with dcSSc at baseline (M0) and 26, 38 and 48 months following either HSCT (E) or CYC treatment (F). (A), (B), (E) and (F) Data represent mean±SD, by paired t-test. CYC, cyclophosphamide; dcSSc, diffuse cutaneous systemic sclerosis; HSCT, haematopoietic stem cell transplant; IGH, immunoglobulin heavy chain.

Extending on our observation that mutation rates following HSCT were decreased, we investigated whether the clonality of the IGH repertoires was affected by transplant not seen after CYC treatment. We analysed the clonality of the IGH repertoires of both healthy individuals and those with dcSSc at baseline and 26, 38 and 48 months following either HSCT or CYC treatment by identifying distinct IGH mRNA sequences (based on the UMI attached to each mRNA sequence) with identical V and J genes, equal CDR3 lengths, and at least 60% amino-acid sequence CDR3 homology. A cluster of sequences meeting these criteria was considered a ‘clonal expression’ if it represented at least 1% of the total repertoire size. Note that this definition of ‘clonal expression’ represents a surrogate for B cell clonality given it counts each distinct IGH mRNA sequence based on its UMI, and thus includes multiple IGH mRNA sequences expressed by individual B cells as well as those expressed by clonally related B cells. Comparison of the clonal expression of the repertoires in healthy individuals and those with dcSSc at baseline yielded no significant differences (online supplemental figure 2A). Next, we analysed the clonal expressions in all patients with dcSSc that had clonal expressions at baseline. Comparison of the clonal expression of the repertoires of dcSSc individuals following either HSCT or CYC revealed a significant decrease in the average clonal expressions of individuals that underwent HSCT at 26 and 48 months following treatment as compared with baseline (figure 2A). In contrast, no significant changes in clonal expressions were observed at any of the timepoints following treatment with CYC (figure 2B). The SI was used as a measure of population heterogeneity in the HSCT and CYC treatment groups (online supplemental file 2B–D).

We further analysed changes in clonality following either HSCT or CYC treatment relative to baseline in individuals for which samples were available from consecutive time points. We observed a trend towards a decrease in clonality in the five individuals that underwent HSCT (figure 2C). In contrast, this trend was not observed among the repertoires of the five individuals treated with CYC for which we had both baseline samples as well as at least one subsequent consecutive time point following treatment (figure 2D). We did not identify significant changes in clonal expression size following HSCT or CYC treatment (figure 2E and F). B cell receptor statistics of patient IGH sequences, with number of clones and percent clonality presented in online supplemental file 2. Our findings suggest that HSCT contributes to a repopulation of a diverse B cell repertoire with a lesser degree of clonality.

### Underusage of IGHV5-51 in patients with dcSSc is normalised following HSCT

**Figure 3:**
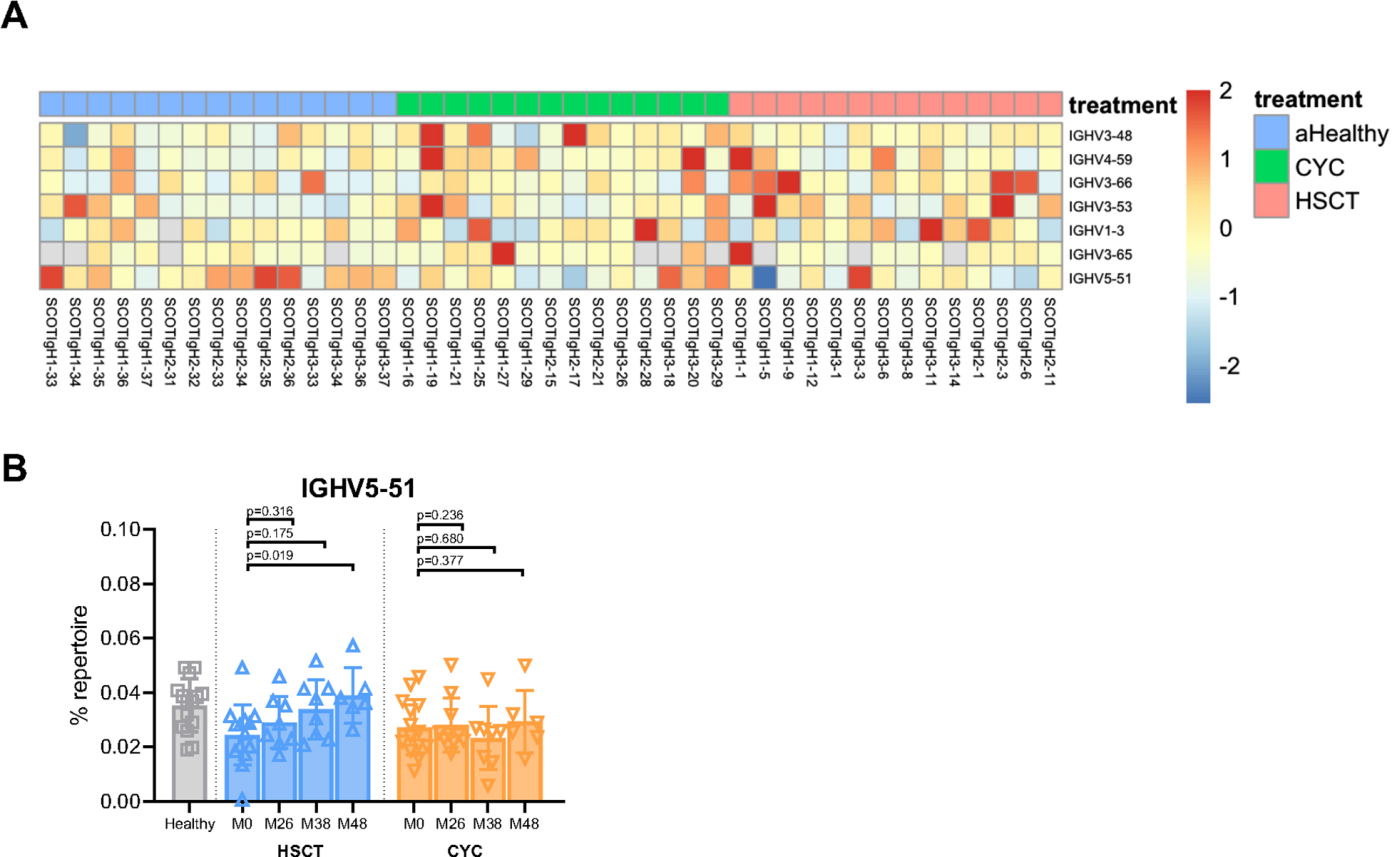
Differences in IGHV gene usage in patients with dcSSc. (A) Heatmap depicting the z-scores of the fraction of usage within each repertoire for the seven genes identified to have significant differences in usage between healthy individuals and individuals with dcSSc at baseline through SAM (q<0.1). (B) Quantification of the per cent of each repertoire using IGHV5-51 in healthy individuals at baseline and in individuals with dcSSc at baseline (M0) and 26, 38 and 48 months following either HSCT or CYC treatment. Data represent mean±SD, by paired t-test. CYC, cyclophosphamide; dcSSc, diffuse cutaneous systemic sclerosis; HSCT, haematopoietic stem cell transplant; IGHV, immunoglobulin heavy chain V gene; SAM, significance analysis of microarrays.

We next investigated whether either HSCT or CYC treatment affected V-gene usage in IGH repertoires of individuals with dcSSc. We first identified V genes with significant differences in usage between healthy individuals and those with dcSSc at baseline by performing SAM.^29^ SAM identified seven IGHV genes that were enriched in dcSSc individuals (figure 3A). Six of these IGHV genes exhibited significantly elevated rates of usage in dcSSc individuals as compared with healthy, whereas one gene, IGHV5-51, exhibited decreased usage in dcSSc individuals as compared with healthy (figure 3A). We analysed the dynamics of usage of each of these seven IGHV genes over time following either HSCT or CYC treatment. We determined the percentage of each individual repertoire comprised of each V gene identified through SAM at baseline and at 26, 38 and 48 months following initiation of either HSCT or CYC treatment. This analysis demonstrated a gradual but significant increase in usage of IGHV5–51, 38 and 48 months following HSCT (figure 3B). In contrast, the other six V genes identified as significant at baseline, IGHV1–3, 3–53, 3–48, 4–59, 3–65 and 3–66, showed no differences in usage over time in either treatment arm (online supplemental figure 3A–F). Our findings indicate dysregulated V gene usage in patients with dcSSc, and also suggest that HSCT affects the dynamics of at least one V gene.

## Discussion

The SCOT clinical trial recently demonstrated that myeloablative autologous HSCT provides significant benefit over CYC for the treatment of individuals with dcSSc.^21^ Here, our analysis of the IGH repertoires of individuals from this trial, undergoing either treatment, revealed key differences that may be informative in understanding how myeloablative autologous HSCT provides benefit, as well as providing additional insights into the dysregulated mechanisms contributing to the pathogenesis of dcSSc. Together, our findings suggest myeloablative autologous HSCT resets the IGH repertoire to a more naïve state, providing a possible mechanism for the elimination of pathogenic B cells that may contribute to the benefit of HSCT over CYC in the treatment of dcSSc.

V(D)J recombination is a defining characteristic of adaptive immunity, and as a result the findings reported here underscore the profound effect of myeloablative conditioning and CD34-selected autologous stem cell transplant on subsequent host adaptive immunity and autoimmunity. Such reprogramming of adaptive immunity may well drive the reported genomic and proteomic corrections noted after HSCT for SSc.^24 31 32^

Our bulk mRNA sequencing analysis of the IGH from PBMCs of individuals with dcSSc following either myeloablative autologous HSCT or CYC treatments revealed an increased proportion of repertoires comprised of the IgM isotype following HSCT. This increase was accompanied by a sustained decrease in mean mutation rate in the IgM fraction following HSCT, but not CYC. These findings suggest that up to 4 years following transplantation, unswitched B cells continue to repopulate the immune system, potentially replacing pathogenic B cells. Our observation is consistent with a previous study of immune reconstitution in patients with dcSSc following non-myeloablative autologous HSCT which demonstrated lower B cell replication in peripheral blood and that an increase in the influx of bone marrow derived naive B cells continues for at least 3 years following transplantation.^8^ Another study similarly demonstrated increased naïve B cells and decreased memory B cells up to a year following myeloablative autologous HSCT in patients with SSc.^33^ Recent transcriptomic studies on blood samples from patients in the SCOT trial demonstrated activation of B cell modules following HSCT, which may arise from an increase in the naïve B cell population.^24^ Further, the persistence of increased IgM expression at later timepoints suggests that transplanted B cells continued to contribute to immune reconstitution up to 4 years post-transplant. As the IgM signatures we detected could be indicative of either naïve or memory B cells, additional studies are needed to definitively identify the source of IgM-expressing B cells bearing the low mutational load and to further parse the role of the increasing IgM populations in dcSSc treatment.

Our analysis demonstrates decreased clonality of the IGH repertoire following HSCT treatment. These findings suggest that repopulation of the B cell repertoire, following transplantation, results in a greater number of distinct clonotypes, rather than the expansion of a few clonal families. As myeloablative autologous HSCT results in the eradication of the existing immune repertoire, it may be expected that an increase in unique clonotypes will be observed for HSCT as compared with CYC treatment, which may only eliminate certain populations of B cells. Dysregulation of B cells has previously been shown in patients with SSc, demonstrating increased levels of circulating naïve B cells, diminished memory B cell populations, the presence of autoantibodies, increased expression of CD19 and infiltration of B cells into the skin and lung tissue of these patients.^3 4 34 35^ While it remains unknown how B cells contribute to the pathogenesis of dcSSc, our findings suggest HSCT treatment results in a B cell repertoire specific for a wider range of antigens which may result in lower targeting of one or multiple pathogenic antigens.

In this study, we report lower usage of IGHV5–51 in patients with dcSSc at baseline as compared with healthy controls. Further, we found that IGHV5–51 usage increased in HSCT patients over time following treatment. IGHV5–51 underusage in dcSSc has not been previously described. A previous study in patients with dcSSc with pulmonary arterial hypertension (PAH) identified lower IGHV2–5 usage in dcSSc-PAH compared with healthy controls. Interestingly, similar to the increase in IGHV5–51 usage in the HSCT arm of the study, the defect in IGHV2–5 usage in patients with dcSSc-PAH was transiently reversed following rituximab mediated B cell depletion.^36^ In addition, a recent study in patients with dcSSc identified higher IGHV3–9 usage relative to controls as well as differential use of several other IGHV and IGHJ usage combinations.^37^

This study has several limitations. (1) Limited sequencing depth may have hampered our ability to identify small clonal families and to comprehensively characterise the gene usage diversity of the IGH repertoires. (2) The longitudinal analysis findings apply only to those healthy enough to complete assessments at weeks 38 or 48. (3) The small number of patients sampled and limited longitudinal data limit the insights into the extent to which IGH repertoires differ between healthy and dcSSc individuals, as well as how dynamic they are following treatment with either HSCT or CYC. A larger study of the full SCOT trial cohort, which would be necessary to provide larger datasets to enable comparison of individuals who responded with those who did not respond to treatment, may provide additional insights into how HSCT and CYC treatment modulate IGH repertoires and its possible clinical relevance. (4) Bulk IGH sequencing precludes our ability to perform functional follow-up studies on the antibodies identified. (5) Bulk IGH sequencing of bulk PBMC RNA is biased to include more sequences from B cells producing large amounts of IGH transcript, such as plasmablasts. Our methods attached a UMI to each distinct IGH mRNA, and thus each copy of mRNA from a single B cell would receive a distinct UMI. Our calculation of % clonal expressions (online supplemental table 2) was based on the number of distinct IGH VDJ CDR3 sequences identified, and thus overrepresents true B cell clonality due to multiple copies of IGH mRNA derived from an individual B cell each receiving a unique UMI and thus being counted multiple times in the calculation of % clonal expressions. (6) Our specific IGH sequencing method only captured IgG, IgA and IgM sequences. (7) The HSCT treatment arm received CYC as part of immunoblative conditional regimen prior to CD34^+^ stem cell transplantation while the CYC treatment arm received monthly infusions of CYC for 1 year, and further studies will be needed to gain a better understanding the impact of CYC, and different CYC dosing regimens, on the B cell repertoire in dcSSc. (8) Analysis of peripheral blood B cells does not capture the effect of HSCT or CYC treatment on local tissue infiltrating B cells or long-lived antibody secreting plasma cells in the bone marrow. Finally, studies are needed to analyse the functional differences in B cell subsets and antibodies present before and after HSCT.

10.1136/ard-2021-221925.supp3 Supplementary data

In summary, sequencing the IGH repertoire of individuals with dcSSc before and after myeloablative autologous HSCT revealed distinct changes following treatment. Following HSCT, we identified an increase in the IgM composition, as well as associated decreases in somatic hypermutation levels. Additionally, the IGH repertoires of individuals undergoing HSCT exhibited decreases in clonality. Together, our findings indicate that myeloablative autologous HSCT results in a resetting of the B cell repertoire that may target a new set of antigens, and may limit the expansion of clones targeting potentially pathogenic antigens. Further, we uncovered novel distinct differences in V gene usage between individuals with dcSSc and healthy individuals. Investigating the dysregulation of these genes may further inform the mechanisms underlying pathogenic B cells in dcSSc.

## Data Availability

Data are available upon reasonable request.
