# Supplementary material for: Myeloablative autologous haematopoietic stem cell transplantation resets the B cell repertoire to a more naïve state in patients with systemic sclerosis
Source: Ann Rheum Dis. 2022 Oct 14;82(3):357–64. doi: 10.1136/ard-2021-221925 (PMC9918657; doi:10.1136/ard-2021-221925)
Supplement: Supplementary data [file ard-2021-221925supp001.pdf]

Supplemental Table.

Treatment	Patient ID	Visit	Sample ID	Outcome	# Clones	% Clonality
HSCT	SCOT2	D0	SCOTIgH1-1	R	2224	58.52
HSCT	SCOT2	M26	SCOTIgH1-2	R	7511	71.9
HSCT	SCOT2	M38	SCOTIgH1-3	R	8512	76.6
HSCT	SCOT2	M48	SCOTIgH1-4	R	10300	74.63
HSCT	SCOT21	D0	SCOTIgH1-9	NR	25351	88.06
HSCT	SCOT21	M26	SCOTIgH1-10	NR	24346	86.88
HSCT	SCOT21	M38	SCOTIgH1-11	NR	22390	90.15
HSCT	SCOT27	D0	SCOTIgH1-12	R	10415	77.31
HSCT	SCOT27	M26	SCOTIgH1-13	R	2020	76.94
HSCT	SCOT27	M38	SCOTIgH1-14	R	13277	72.13
HSCT	SCOT27	M48	SCOTIgH1-15	R	16158	71.11
HSCT	SCOT3	D0	SCOTIgH1-5	NR	275	94.1
HSCT	SCOT32	D0	SCOTIgH3-1	NR	5289	95.69
HSCT	SCOT32	M26	SCOTIgH3-2	NR	13591	88.56
HSCT	SCOT33	D0	SCOTIgH3-3	R	2963	69.25
HSCT	SCOT33	M38	SCOTIgH3-4	R	10624	69.01
HSCT	SCOT33	M48	SCOTIgH3-5	R	12450	72.1
HSCT	SCOT37	D0	SCOTIgH3-6	NR	7986	73.67
HSCT	SCOT42	D0	SCOTIgH3-8	R	8599	90.71
HSCT	SCOT42	M26	SCOTIgH3-9	R	7110	88.69
HSCT	SCOT42	M48	SCOTIgH3-10	R	4070	76.53
HSCT	SCOT45	D0	SCOTIgH3-11	R	8283	91.01
HSCT	SCOT45	M38	SCOTIgH3-12	R	10716	89.48
HSCT	SCOT45	M48	SCOTIgH3-13	R	14941	83.35
HSCT	SCOT48	D0	SCOTIgH3-14	NR	5737	85.75
HSCT	SCOT48	M26	SCOTIgH3-15	NR	5867	74.09
HSCT	SCOT48	M38	SCOTIgH3-16	NR	8231	85.42
HSCT	SCOT48	M48	SCOTIgH3-17	NR	11590	74.93
HSCT	SCOT51	D0	SCOTIgH2-1	NR	3094	68.61
HSCT	SCOT53	D0	SCOTIgH2-3	NR	3711	66.59
HSCT	SCOT53	M26	SCOTIgH2-4	NR	22459	83.37
HSCT	SCOT53	M38	SCOTIgH2-5	NR	13408	81.21
HSCT	SCOT58	D0	SCOTIgH2-6	NR	12760	93.56
HSCT	SCOT71	M38	SCOTIgH2-9	NR	12691	90.16
HSCT	SCOT71	M48	SCOTIgH2-10	NR	9479	81.27
HSCT	SCOT74	D0	SCOTIgH2-11	R	9624	89.75
HSCT	SCOT74	M26	SCOTIgH2-12	R	16070	92.38
HSCT	SCOT74	M38	SCOTIgH2-13	R	15959	91.44
HSCT	SCOT74	M48	SCOTIgH2-14	R	12821	89.42
CYC	SCOT12	D0	SCOTIgH1-25	NR	8248	87.55
CYC	SCOT18	D0	SCOTIgH1-27	NR	4524	92.88
CYC	SCOT25	D0	SCOTIgH1-29	NR	6500	97.14
CYC	SCOT25	M26	SCOTIgH1-30	NR	3494	95.82
CYC	SCOT25	M38	SCOTIgH1-31	NR	935	91.04
CYC	SCOT25	M48	SCOTIgH1-32	NR	2508	90.98
CYC	SCOT26	D0	SCOTIgH2-15	NR	4442	69.15
CYC	SCOT26	M26	SCOTIgH2-16	NR	12044	71.11
CYC	SCOT41	D0	SCOTIgH2-17	R	9095	97.39
CYC	SCOT41	M26	SCOTIgH2-18	R	5471	86.73
CYC	SCOT41	M38	SCOTIgH2-19	R	5236	80.1
CYC	SCOT41	M48	SCOTIgH2-20	R	6914	84.36
CYC	SCOT43	D0	SCOTIgH2-21	NR	6920	81.4
CYC	SCOT43	M26	SCOTIgH2-22	NR	7067	93.47
CYC	SCOT43	M38	SCOTIgH2-23	NR	241	95.78
CYC	SCOT47	D0	SCOTIgH3-26	NR	6992	76.56
CYC	SCOT47	M26	SCOTIgH3-27	NR	4609	61.15
CYC	SCOT47	M38	SCOTIgH3-28	NR	5586	73.27
CYC	SCOT5	D0	SCOTIgH1-16	R	4943	80.9
CYC	SCOT5	M26	SCOTIgH1-17	R	6220	81.54
CYC	SCOT5	M38	SCOTIgH1-18	R	3117	63.13
CYC	SCOT50	M26	SCOTIgH2-27	NR	7998	81.9
CYC	SCOT50	M38	SCOTIgH2-25	NR	10580	73.15
CYC	SCOT50	M48	SCOTIgH2-26	NR	8481	69.84
CYC	SCOT63	D0	SCOTIgH2-28	NR	1921	90.64
CYC	SCOT63	M26	SCOTIgH2-29	NR	1689	97.33
CYC	SCOT63	M48	SCOTIgH2-30	NR	1041	80
CYC	SCOT64	D0	SCOTIgH3-20	NR	5239	33.5
CYC	SCOT67	M26	SCOTIgH3-21	NR	13269	82.88
CYC	SCOT67	M38	SCOTIgH3-22	NR	10794	72.63
CYC	SCOT67	M48	SCOTIgH3-23	NR	24974	81.69
CYC	SCOT70	M26	SCOTIgH3-25	NR	5607	84.13
CYC	SCOT73	D0	SCOTIgH3-29	R	5909	63.58
CYC	SCOT73	M26	SCOTIgH3-30	R	5033	61.41
CYC	SCOT73	M38	SCOTIgH3-31	R	4954	53.49
CYC	SCOT73	M48	SCOTIgH3-32	R	3649	54.46
CYC	SCOT8	D0	SCOTIgH1-19	NR	4227	92.72
CYC	SCOT9	D0	SCOTIgH1-21	R	4094	80.55
CYC	SCOT9	M26	SCOTIgH1-22	R	7137	79.94
CYC	SCOT9	M38	SCOTIgH1-23	R	7195	85.11
CYC	SCOT9	M48	SCOTIgH1-24	R	7878	84.19
Healthy	HC1	D0	SCOTIgH1-33	Healthy	4974	73.98
Healthy	HC10	D0	SCOTIgH2-35	Healthy	9575	91.43
Healthy	HC11	D0	SCOTIgH2-36	Healthy	10150	87.11
Healthy	HC12	D0	SCOTIgH3-33	Healthy	7636	86.59
Healthy	HC13	D0	SCOTIgH3-34	Healthy	1114	59.41
Healthy	HC14	D0	SCOTIgH3-35	Healthy	5716	65.29
Healthy	HC15	D0	SCOTIgH3-36	Healthy	10006	88.12
Healthy	HC2	D0	SCOTIgH1-34	Healthy	2386	74.16
Healthy	HC3	D0	SCOTIgH1-35	Healthy	4605	61.81
Healthy	HC4	D0	SCOTIgH1-36	Healthy	9274	69.76
Healthy	HC5	D0	SCOTIgH1-37	Healthy	7264	88.3
Healthy	HC6	D0	SCOTIgH2-31	Healthy	3482	82.28
Healthy	HC7	D0	SCOTIgH2-32	Healthy	4040	68.67
Healthy	HC8	D0	SCOTIgH2-33	Healthy	5759	82.18
Healthy	HC9	D0	SCOTIgH2-34	Healthy	3857	73.51

# Clones represents the number of distinct Ig BCR heavy chain VDJ CDR3 sequences obtained.

% Clonality represents the percent of sequences with matched V-J genes and >60% CDR3 homology.
