# Supplementary figures and images for: Myeloablative autologous haematopoietic stem cell transplantation resets the B cell repertoire to a more naïve state in patients with systemic sclerosis
Source: Ann Rheum Dis. 2022 Oct 14;82(3):357–64. doi: 10.1136/ard-2021-221925 (PMC9918657; doi:10.1136/ard-2021-221925)

## Supplemental Figure 1

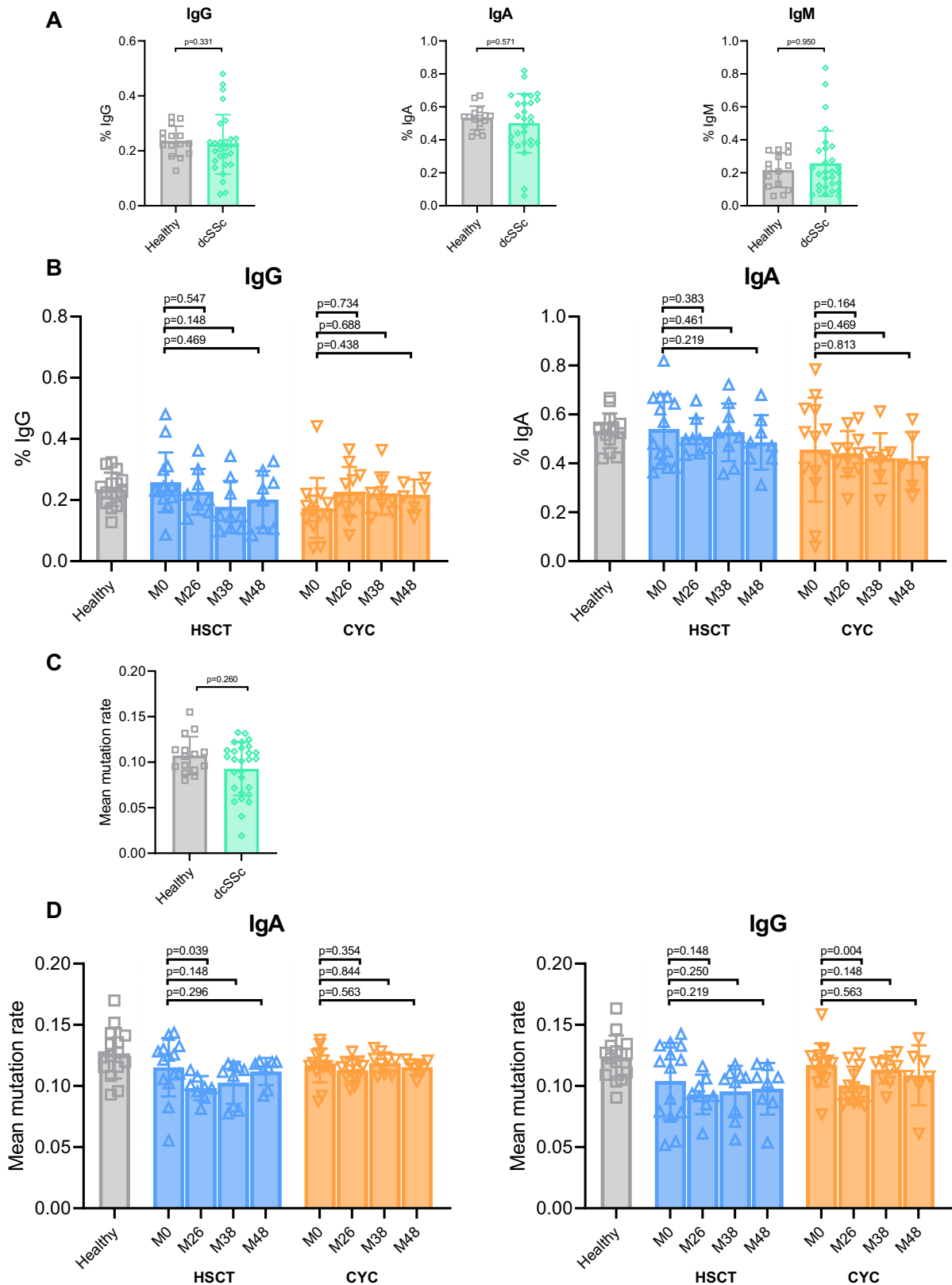

## Supplemental Figure 2

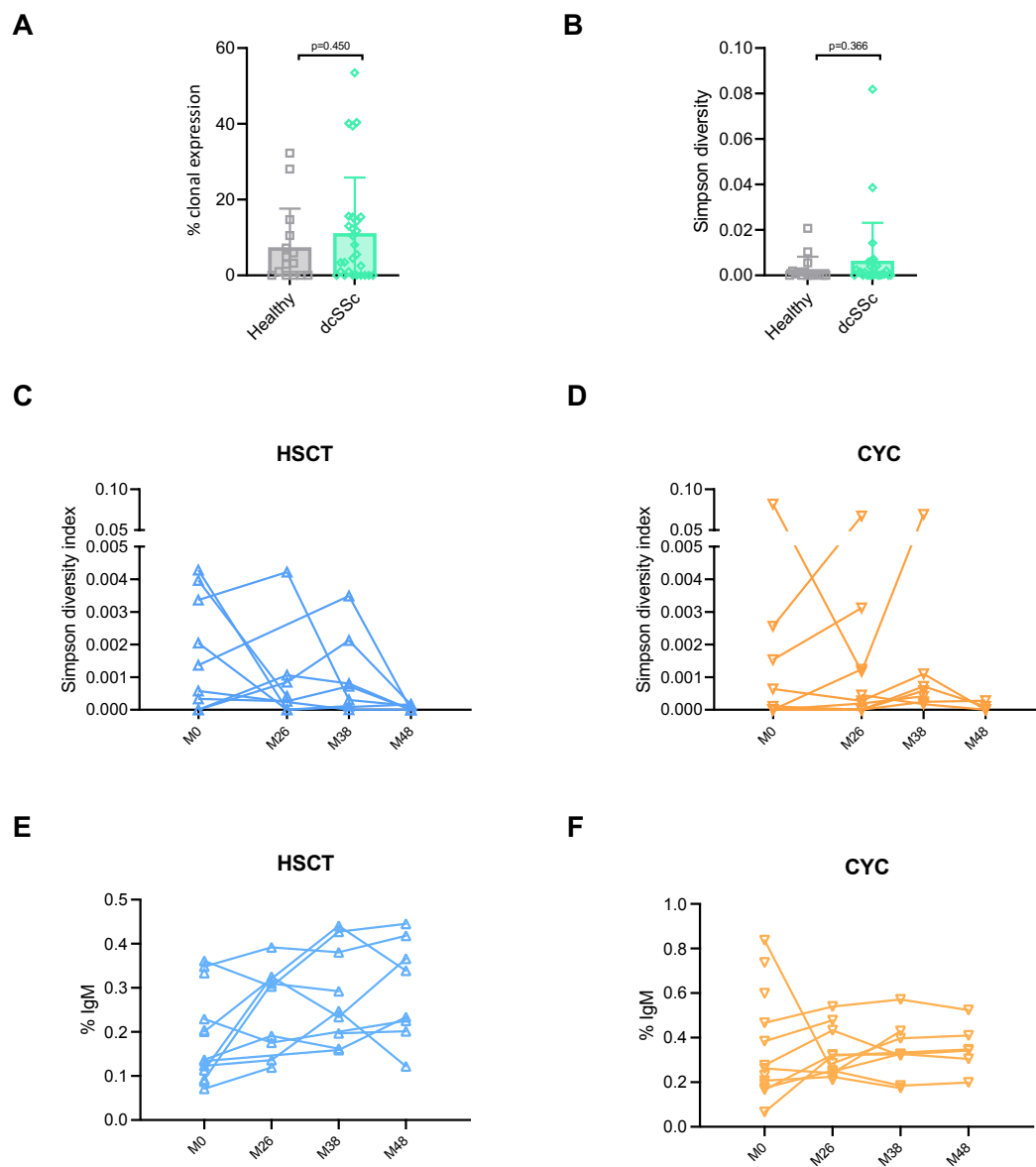

## Supplemental Figure 3

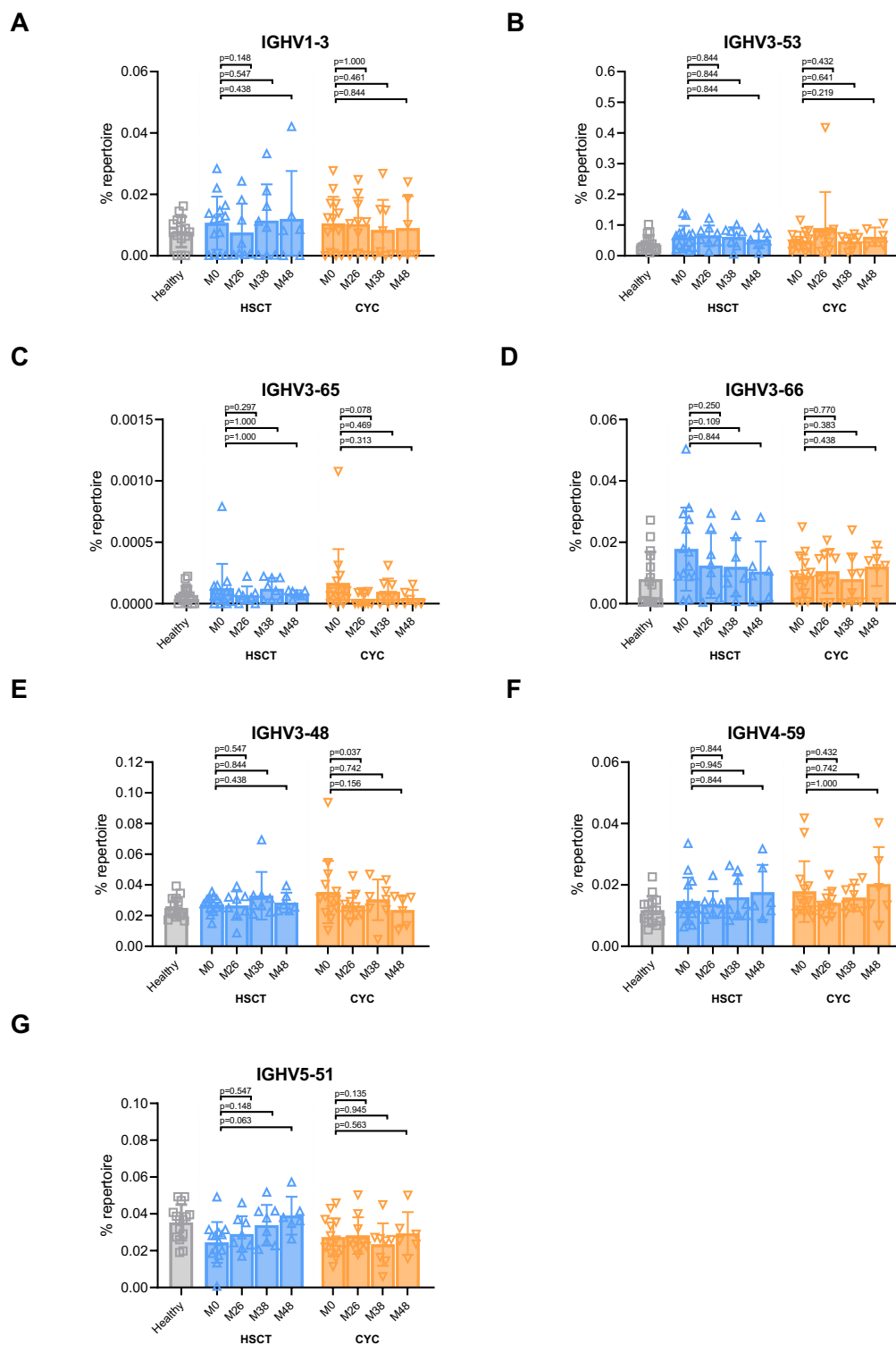

Supplement: Supplementary data [file ard-2021-221925supp002.pdf]
